# Combinations of Host Biomarkers Predict Mortality among Ugandan Children with Severe Malaria: A Retrospective Case-Control Study

**DOI:** 10.1371/journal.pone.0017440

**Published:** 2011-02-25

**Authors:** Laura K. Erdman, Aggrey Dhabangi, Charles Musoke, Andrea L. Conroy, Michael Hawkes, Sarah Higgins, Nimerta Rajwans, Kayla T. Wolofsky, David L. Streiner, W. Conrad Liles, Christine M. Cserti-Gazdewich, Kevin C. Kain

**Affiliations:** 1 S.A. Rotman Laboratories, McLaughlin-Rotman Centre for Global Health, Toronto General Hospital-University Health Network, University of Toronto, Toronto, Ontario, Canada; 2 Makerere University College of Health Sciences, Kampala, Uganda; 3 School of Medicine, University of Queensland, Brisbane, Queensland, Australia; 4 Department of Psychiatry, University of Toronto, Toronto, Ontario, Canada; 5 Tropical Disease Unit, Division of Infectious Diseases, Department of Medicine, Toronto General Hospital, Toronto, Ontario, Canada; 6 Laboratory Medicine Program (Transfusion Medicine), University Health Network/University of Toronto, Toronto, Ontario, Canada; Lile 2 University, France

## Abstract

**Background:**

Severe malaria is a leading cause of childhood mortality in Africa. However, at presentation, it is difficult to predict which children with severe malaria are at greatest risk of death. Dysregulated host inflammatory responses and endothelial activation play central roles in severe malaria pathogenesis. We hypothesized that biomarkers of these processes would accurately predict outcome among children with severe malaria.

**Methodology/Findings:**

Plasma was obtained from children with uncomplicated malaria (n = 53), cerebral malaria (n = 44) and severe malarial anemia (n = 59) at time of presentation to hospital in Kampala, Uganda. Levels of angiopoietin-2, von Willebrand Factor (vWF), vWF propeptide, soluble P-selectin, soluble intercellular adhesion molecule-1 (ICAM-1), soluble endoglin, soluble FMS-like tyrosine kinase-1 (Flt-1), soluble Tie-2, C-reactive protein, procalcitonin, 10 kDa interferon gamma-induced protein (IP-10), and soluble triggering receptor expressed on myeloid cells-1 (TREM-1) were determined by ELISA. Receiver operating characteristic (ROC) curve analysis was used to assess predictive accuracy of individual biomarkers. Six biomarkers (angiopoietin-2, soluble ICAM-1, soluble Flt-1, procalcitonin, IP-10, soluble TREM-1) discriminated well between children who survived severe malaria infection and those who subsequently died (area under ROC curve>0.7). Combinational approaches were applied in an attempt to improve accuracy. A biomarker score was developed based on dichotomization and summation of the six biomarkers, resulting in 95.7% (95% CI: 78.1–99.9) sensitivity and 88.8% (79.7–94.7) specificity for predicting death. Similar predictive accuracy was achieved with models comprised of 3 biomarkers. Classification tree analysis generated a 3-marker model with 100% sensitivity and 92.5% specificity (cross-validated misclassification rate: 15.4%, standard error 4.9%).

**Conclusions:**

We identified novel host biomarkers of pediatric severe and fatal malaria (soluble TREM-1 and soluble Flt-1) and generated simple biomarker combinations that accurately predicted death in an African pediatric population. While requiring validation in further studies, these results suggest the utility of combinatorial biomarker strategies as prognostic tests for severe malaria.

## Introduction

*Plasmodium falciparum* malaria causes almost one million deaths annually, mostly among young children in sub-Saharan Africa [1]. The most common manifestations of pediatric severe malaria are severe malarial anemia (SMA) and cerebral malaria (CM). These syndromes can have case fatality rates as high as 20% [2]. It is challenging at clinical presentation to accurately determine which children with severe malaria are at greatest risk of death. Simple and sensitive clinical scores have been developed to predict outcome, but they have low specificity and rely on subjective assessment of clinical signs [3], [4]. An accurate prognostic test would be useful for targeting limited health resources to high-risk children and for selecting patients to enroll in clinical trials of adjunctive therapies for severe malaria.

Investigations into malaria pathogenesis have implicated host pathways in disease progression. In particular, dysregulated inflammatory responses and endothelial activation are thought to be central processes in severe malaria pathogenesis [5]–[7]. We hypothesized that plasma biomarkers of these pathways may have clinical utility as prognostic tools, particularly if used in combination. We examined biomarkers of these pathways for their utility as indicators of disease severity and outcome in Ugandan children presenting to hospital with malaria.

Excessive pro-inflammatory responses to infection are observed in both CM and SMA [8]–[11]. In this study, we measured plasma levels of acute-phase response components, C-reactive protein (CRP) and procalcitonin (PCT), which have been shown to increase during malaria infection [12], [13]. We also measured 10 kDa interferon gamma-induced protein (IP-10), a chemokine reported to be elevated in fatal CM [14]. Moreover, we assessed levels of soluble triggering receptor expressed on myeloid cells-1 (sTREM-1), which is associated with inflammatory conditions [15] but has not been previously investigated in malaria.

Dysregulated inflammation is thought to promote CM in part through endothelial activation in the brain. Pro-inflammatory cytokines upregulate cell adhesion receptors (e.g., intercellular adhesion molecule-1 [ICAM-1]) that mediate sequestration of parasitized erythrocytes in brain microvasculature, leading to vessel occlusion [16] and blood-brain barrier dysfunction [17]. Upon endothelial activation, soluble endothelial cell receptors are released via ectodomain shedding or alternative splicing. We measured the soluble forms of ICAM-1 (sICAM-1) and the TGF-β receptor endoglin (s-endoglin), which have both been shown to be increased in severe malaria [18], [19], and soluble FMS-like tyrosine kinase-1 (sFlt-1), which has been implicated in placental malaria [20]. Endothelial activation also causes exocytosis of Weibel-Palade bodies (WPB), intracellular vesicles that contain a variety of effector molecules [21]. We assayed WBP-associated factors angiopoietin-2 (Ang-2), von Willebrand factor (vWF), vWF propeptide, and soluble P-selectin (sP-selectin). Some of these molecules are elevated in CM [22]–[24] and have been suggested to contribute to pathology: Ang-2 may exacerbate vascular activation in malaria by antagonizing the quiescence-promoting interaction of the endothelial Tie-2 receptor with angiopoietin-1 (Ang-1) [25], while vWF may help tether parasitized erythrocytes to endothelial cells via platelets [26]. In addition to CM patients, systemic endothelial activation has been shown to occur in adults with uncomplicated and non-CM severe malaria [19], [27]; however, few studies have characterized the extent and significance of this process in pediatric SMA.

In this study, we examined plasma biomarkers of inflammation and endothelial activation in children presenting to hospital with malaria. We determined which markers were elevated in severe disease compared to uncomplicated malaria (UM), and which markers discriminated between children who survived severe malaria infection and those who subsequently died. Furthermore, we identified combinations of biomarkers from these two host pathways that accurately predicted mortality among children with severe malaria.

## Methods

### Ethics statement

Ethical approval for the study was obtained from the Mulago Hospital Research Ethics Committee, Makerere University Faculty of Medicine Research Ethics Committee, Uganda National Council for Science & Technology, and the University Health Network. Written informed consent was obtained from parents/guardians before enrollment.

### Study site and participants

This retrospective case-control study was nested within a larger study conducted at Mulago Hospital in Kampala, Uganda between October 2007 and October 2009. Mulago Hospital is a national referral hospital that serves Kampala and surrounding districts. Malaria transmission in this region and the patient population at Mulago Hospital have been previously described [28]. Children presenting to hospital were eligible for enrollment if they were between 6 months and 12 years old and had microscopy-confirmed *P. falciparum* infection (asexual parasitemia with clinical signs or symptoms of malaria). Children were excluded if they had sickle cell trait/disease, HIV co-infection, or severe malnutrition. Clinical and demographic data were collected upon enrollment, and venous blood samples were collected for routine measurement of hemoglobin and platelet count, and for plasma banking. Thin blood smears were obtained at presentation for determination of parasitemia, which is reported as the arithmetic mean of two independent readings by expert microscopists. Treatment was in accordance with Ugandan national guidelines: artemether/lumefantrine was administered to children with uncomplicated malaria, and parenteral quinine was used in severe malaria cases [29]. All children with SMA received blood transfusions. Children were followed for recovery/survival or death.

For biomarker analysis, a sub-group (n = 156) of UM outpatients, CM inpatients, and SMA inpatients in roughly equal numbers was selected from the larger study based on availability of an adequate volume of previously unthawed plasma. CM was defined as an unrousable coma (not attributable to any other cause) in a child with asexual *P. falciparum* parasitemia (i.e. Blantyre Coma Scale score <3, either before or >6 h after seizures or anticonvulsant medication (if applicable), or repeated (>3) seizures witnessed within a 24 h period, in the absence of hypoglycaemia (<40 mg/dL or 2.2 mM) or any known alternative neurologic abnormalities). SMA was defined as hematocrit<15% or hemoglobin<5.0 g/dL in the presence of asexual parasitemia.

### Biomarker assays

Plasma (sodium citrate anticoagulant) was stored at −20°C prior to testing. ELISAs were used to quantify plasma biomarker levels and were performed blinded to all associated clinical data. The following markers were assayed (dilution factor indicated in parentheses): Ang-2 (1∶5), CRP (1∶40,000), sTREM-1 (neat), s-endoglin (1∶25), IP-10 (1∶2), sFlt-1 (1∶6), sICAM-1 (1∶1000), sP-selectin (1∶50), sTie-2 (1∶25; all R&D Systems), PCT (1∶5; Ray BioTech), and vWF propeptide (1∶400; Sanquin). ELISAs were performed according to the manufacturers' instructions, with the following changes: assays were performed in a volume of 50 µL/well; plasma samples were incubated overnight at 4°C; and ELISAs were developed using Extravidin®-Alkaline Phosphatase (Sigma, 1∶1000 dilution, 45 min incubation) followed by addition of p-Nitrophenyl phosphate substrate (Sigma) and optical density readings at 405 nm. s-endoglin and sP-selectin were only measured in samples collected during the first year of the study (n = 101). For vWF, plates were coated with anti-human vWF antibody (Dako, 1∶600), incubated with samples (1∶500 dilution) and serial dilutions of recombinant vWF (American Diagnostica), then incubated with horseradish peroxidase-conjugated anti-human vWF (Dako, 1∶8000). Assays were developed with tetramethylbenzidine, stopped with H_2_SO_4_, and read at 450 nm. Background signal was determined from blank wells included on each plate (assay buffer added instead of sample), and background optical density was subtracted from all samples and standards prior to analysis. Samples with optical densities below the lowest detectable standard were assigned the value of that standard.

### Statistical analysis

GraphPad Prism v4, SPSS v18, and MedCalc software were used for analysis. For clinical and demographic variables, differences between groups were assessed using the Chi-square test (categorical variables) or the Kruskal-Wallis test with Dunn's multiple comparison post-hoc tests (continuous variables). The Mann-Whitney U test was used to compare biomarker levels between groups, and p values were corrected for multiple comparisons using Holm's correction. Receiver operating characteristic curves were generated using the non-parametric method of Delong et. al [30]. Cut-points were determined using the Youden index (J = max[sensitivity+specificity−1]). For logistic regression, linearity of an independent variable with the log odds of the dependent was assessed by including a Box-Tidwell transformation into the model and ensuring that this term was not significant. Bootstrapping (1000 sample draws) was used to generate variance estimates for b. Model goodness-of-fit was assessed by the Hosmer-Lemeshow test and calibration slope analysis [31]. Positive and negative predictive values were calculated using the reported case fatality rate of 5.7% for microscopy-confirmed CM and SMA at Mulago Hospital [28]. Classification tree analysis was performed in SPSS with the following settings: minimum 10 cases for parent nodes and 5 for child nodes; customized prior probabilities based on the case fatality rate at Mulago Hospital; customized misclassification costs (as indicated); pruning to reduce overfitting; and cross-validation with 10 sample folds to generate an estimate of the misclassification rate and its standard error. There were no missing values from the dataset.

## Results

### Characteristics of study participants

Children presenting to Mulago Hospital in Kampala, Uganda with UM (n = 53), CM (n = 44), and SMA (n = 59) were included in the study. Six children had concurrent CM and SMA and were categorized as “CM”, and five children with SMA exhibited decreased consciousness but did not meet study criteria for CM. Table 1 presents the demographic and clinical characteristics of the three groups. Children with SMA were younger than children with UM and CM (p<0.001) and presented significantly later than the other groups (p<0.001, approximately one day later). Children with severe malaria had lower hemoglobin levels and platelet counts than children with UM.

**Table 1:** Demographic and clinical characteristics of study participants presenting with uncomplicated and severe malaria.^a^

				Pooled severe malaria	
Characteristic	UM^b^ (n = 53)	CM^c^ (n = 44)	SMA (n = 59)	Survivors (n = 80)	Fatalities (n = 23)
Gender (% female)	45.3	52.3	49.2	46.3	65.2§§
Age (years)	4.4 (2.1, 8.1)	3.0 (1.5, 4.3)	1.3 (0.9, 2.0)*** ###	1.6 (1.0, 3.1)	1.9 (1.2, 3.3)
Days reported ill prior to presentation	3 (2, 4)	3 (2, 4)	4 (3, 5)*** #	3 (3, 4)	3 (2, 7)
Parasitemia (parasites/uL)	3.8×10^4^ (1.6×10^4^, 1.2×10^5^)	9.8×10^4^ (1.5×10^4^, 2.7×10^5^)	2.6×10^4^ (7.4×10^3^, 1.2×10^5^)#	3.7×10^4^ (7.5×10^3^, 1.5×10^5^)	1.6×10^5^ (2.2×10^4^, 3.9×10^5^)§
Hemoglobin (g/dL)	10.1 (9.4, 11.3)	6.3 (5. 3, 8.4)***	3.8 (3.2, 4.4)*** ###	4.3 (3.4, 5.6)	5.4 (4.2, 8.3)§ ^,^ d
Platelet count (×10^9^/L)	166 (107, 219)	73 (47, 128)***	116 (71, 165)*	103 (61, 162)	73 (41, 128)
Fatal cases	0	14	9	0	23

a All variables except gender are presented as median (interquartile range). Groups were compared using the Mann Whitney U test or Kruskal-Wallis test with Dunn's post-hoc tests (continuous variables) or Chi-square test (categorical variables).

b UM, uncomplicated malaria; CM, cerebral malaria; SMA, severe malaria anemia.

c 6 children with concurrent CM and SMA were included in the CM group. 5 children with SMA exhibited decreased consciousness but did not meet criteria for CM.

d Increased hemoglobin among fatalities was due to the higher CM∶SMA ratio in this group vs survivors.

*p<0.05,

***p<0.001 CM or SMA vs. UM.

# p<0.05,

### p<0.001 SMA vs. CM.

§ p<0.05,

§§ p<0.01 fatalities vs. survivors.

### Biomarker levels in uncomplicated vs. severe malaria patients

**Figure 1:**
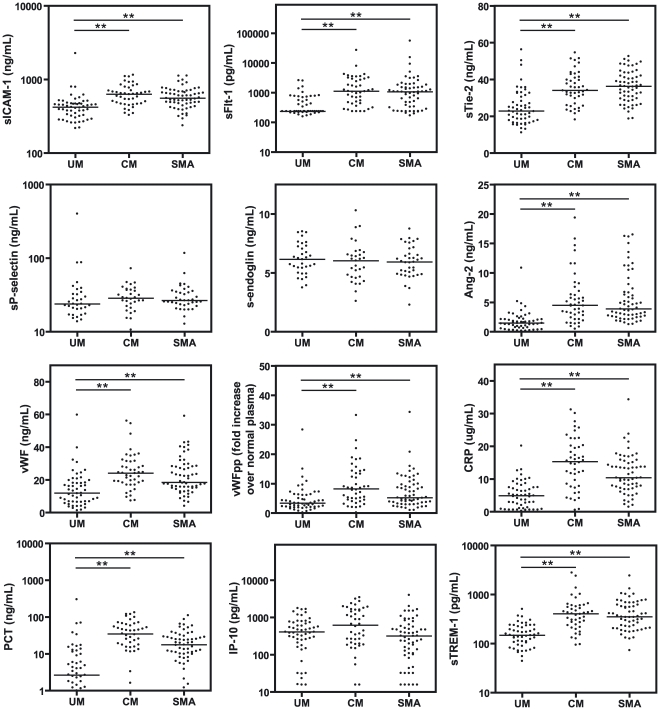
Plasma biomarker levels in Ugandan children with uncomplicated and severe malaria at time of presentation. Biomarkers of inflammation and endothelial activation in the plasma of children with uncomplicated malaria (UM), cerebral malaria (CM), and severe malarial anemia (SMA) were measured by ELISA. Data are presented as dot plots with medians. A Mann Whitney U test was performed for each comparison, and p values were adjusted for multiple comparisons using Holm's correction (n = 24). ** p<0.01.

Plasma samples obtained at presentation were assayed for biomarkers of endothelial activation and inflammation (Fig. 1). sICAM-1, sTie-2, and sFlt-1 were significantly increased in CM and SMA compared to UM (p<0.01), while s-endoglin, sP-selectin, and IP-10 did not differ between groups (p>0.05). WPB-associated proteins Ang-2, vWF, and vWF propeptide were elevated in children with severe malaria compared to UM, as were inflammatory biomarkers CRP, PCT, and sTREM-1 (p<0.01).

### Biomarkers as predictors of mortality in children with severe malaria

**Figure 2:**
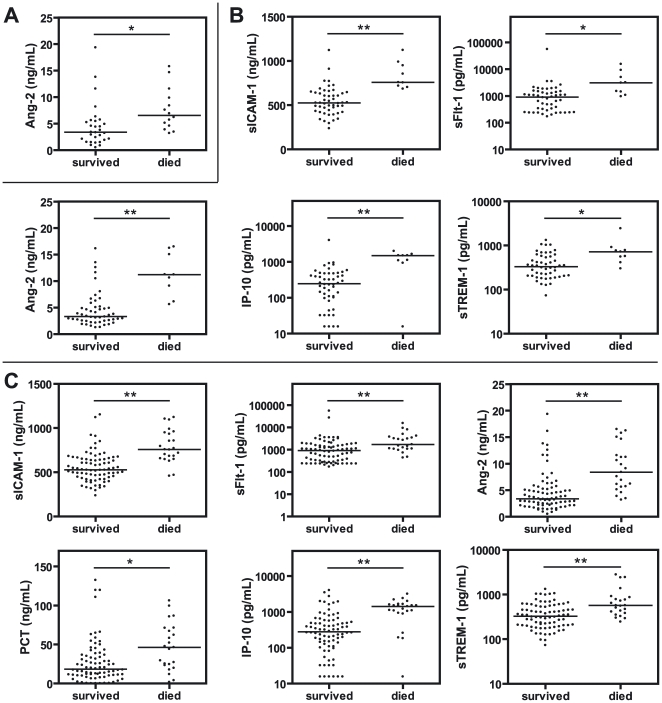
Plasma biomarker levels in children with severe malaria who survived or subsequently died from infection. Presented are biomarkers that were significantly different for (A) CM patients only, (B) SMA patients only, and (C) all severe malaria patients combined. Biomarkers were measured by ELISA. Data are presented as dot plots with medians. A Mann Whitney U test was performed for each comparison, and p values were adjusted for multiple comparisons using Holm's correction (n = 12 for each group). * p<0.05 and ** p<0.01.

To evaluate the prognostic utility of these plasma biomarkers, we compared admission levels between children with severe malaria who survived infection and those who subsequently died. After correction for multiple comparisons, admission levels of Ang-2 were significantly increased in CM fatalities compared to survivors (Fig. 2A; p<0.05), while Ang-2, sICAM-1, IP-10 (p<0.01), sTREM-1 and sFlt-1 (p<0.05) were elevated in SMA fatalities compared to survivors (Fig. 2B).

The biomarkers that reached significance in the SMA group but not the CM group after correction for multiple comparisons (sICAM-1, IP-10, sTREM-1, sFlt-1) were significant or trending towards significance in the CM group before the correction was applied. This suggests that the apparent differences between syndromes may have been due to low statistical power and therefore we combined all severe malaria patients for further analysis. This strategy also avoids the problem of classifying mixed clinical phenotypes, as occurred in the present study population. Characteristics of survivors and fatalities were similar (Table 1), although among fatalities there was a greater proportion of females (p = 0.007) and increased parasitemia (p = 0.023). We found that Ang-2, sICAM-1, sFlt-1, IP-10, and sTREM-1 (p<0.01), as well as PCT (p<0.05), were elevated in fatal cases of severe malaria compared to survivors (Fig. 2C).

**Figure 3:**
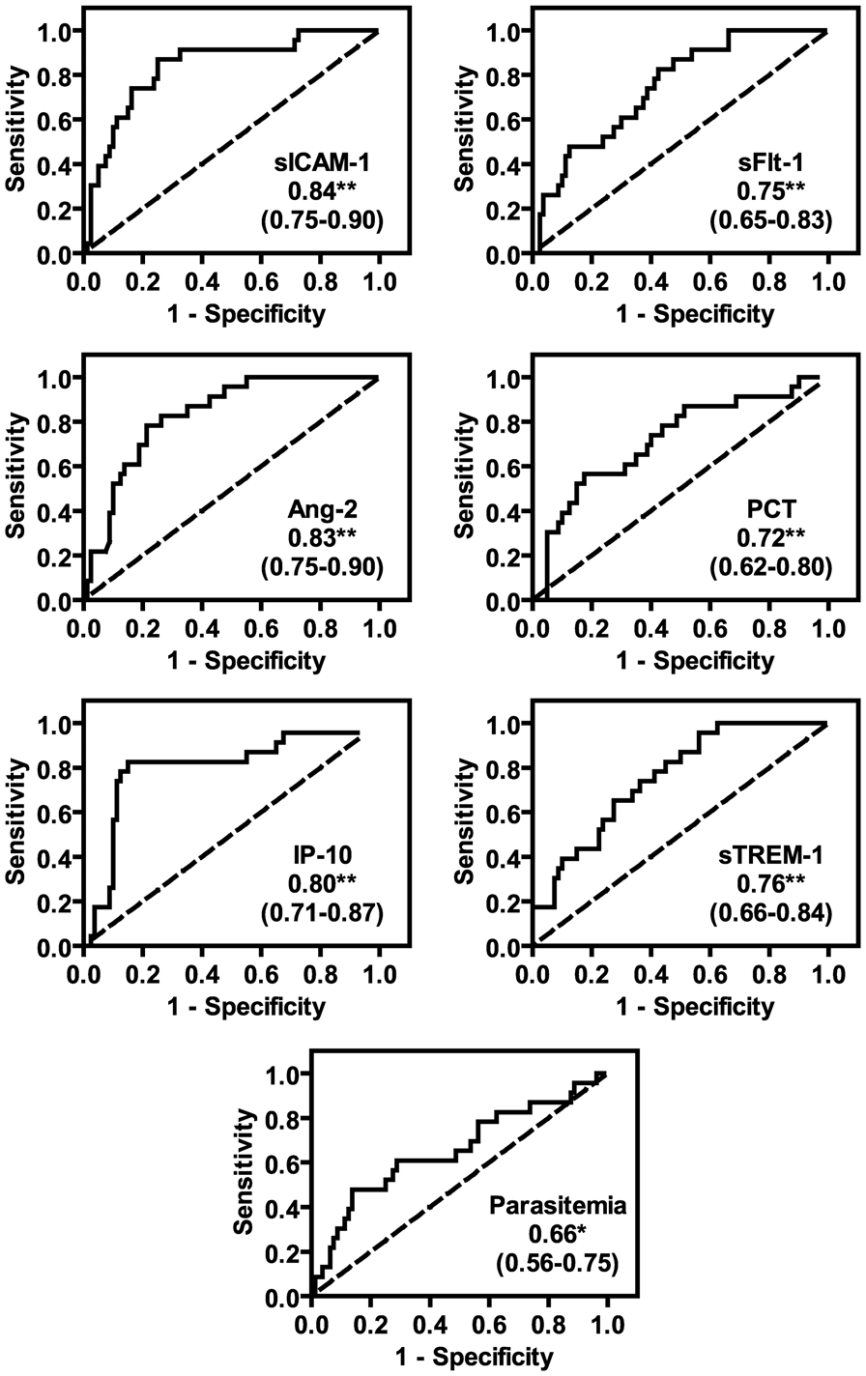
Assessment of biomarker utility in predicting outcome in children with severe malaria. A receiver operating characteristic (ROC) curve was generated for each biomarker. The dashed reference line represents the ROC curve for a test with no discriminatory ability. Area under the ROC curve is displayed on each graph with 95% confidence intervals in parentheses. p values were adjusted for multiple comparisons using Holm's correction (n = 7). * p<0.05 and ** p<0.01.

To assess how well these biomarkers discriminated between survivors and fatalities, we generated receiver operating characteristic (ROC) curves and determined area under the curve (AUC) (Fig. 3). Ang-2, sICAM-1, and IP-10 had excellent predictive ability (AUC 0.8–0.9), and sTREM-1, sFlt-1 and PCT had acceptable predictive ability (AUC 0.7–0.8) [32]. The AUC for parasitemia, which is used in clinical practice as a prognostic factor [33], was 0.66.

We used the Youden index to obtain a cut-point for each biomarker, and evaluated clinical performance measures for these dichotomized biomarkers (Table 2). sTREM-1 achieved the highest sensitivity (95.7%) but had low specificity (43.8%), while IP-10 predicted death with the highest overall accuracy (82.6% sensitivity, 85% specificity).

**Table 2:** Clinical performance of biomarkers for predicting mortality among children with severe malaria.^a^

Biomarker	Cut-point^b^	Sensitivity (%)	Specificity (%)	PLR^c^	NLR	PPV (%)^d^	NPV (%)
Ang-2	>5.6 ng/mL	78.3 (56.3–92.5)	78.8 (68.2–87.1)	3.7 (2.9–4.7)	0.3 (0.1–0.7)	18.2 (5.8–38.7)	98.4 (92.4–99.9)
sICAM-1	>645.3 ng/mL	87.0 (66.4–97.2)	75.0 (64.1–84.0)	3.5 (2.8–4.3)	0.2 (0.06–0.5)	17.4 (5.9–35.9)	99.0 (93.2–100)
sFlt-1	>1066.3 pg/mL	82.6 (61.2–95.0)	57.5 (45.9–68.5)	1.9 (1.5–2.5)	0.3 (0.1–0.8)	10.5 (3.4–23.1)	98.2 (90.4–100)
PCT	>43.1 ng/mL	56.5 (34.5–76.8)	82.5 (72.4–90.1)	3.2 (2.2–4.7)	0.5 (0.3–1.0)	16.3 (3.8–39.5)	96.9 (90.5–99.5)
IP-10	>831.2 pg/mL	82.6 (61.2–95.0)	85.0 (75.3–92.0)	5.5 (4.5–6.8)	0.2 (0.07–0.6)	25.0 (8.3–49.8)	98.8 (93.4–100)
sTREM-1	>289.9 pg/mL	95.7 (78.1–99.9)	43.8 (32.7–55.3)	1.7 (1.3–2.2)	0.1 (0.01–0.7)	9.3 (3.3–19.6)	99.4 (90.5–100)

a All parameters are presented with 95% CIs in parentheses.

b Cut-points were determined using the Youden Index (J = max[sensitivity+specificity−1]).

c PLR, positive likelihood ratio; NLR, negative likelihood ratio; PPV, positive predictive value; NPV, negative predictive value.

d PPVs and NPVs were based on estimates that 5.7% of CM and SMA patients at Mulago hospital die of the malaria infection [28].

### Predicting mortality using a “biomarker score”

We hypothesized that combining biomarkers would improve predictive accuracy. The modest number of deaths in the study precluded multivariable logistic regression analysis with more than 2–3 independent variables [34]. Therefore, as performed in other conditions [35], [36], we combined the biomarkers into a score. For each marker, one point was assigned if the measured value was greater than the corresponding cut-point, and zero points were assigned if it was lower. A cumulative “biomarker score” was calculated for each patient by summing the points for all six markers. No two dichotomized biomarkers were highly correlated (Spearman's rho<0.6; data not shown), suggesting that each biomarker would contribute unique information to the score.

**Figure 4:**
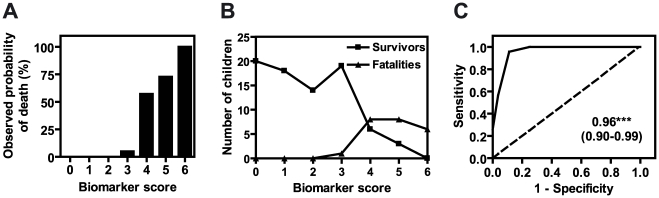
The biomarker score is significantly associated with risk of fatality among children with severe malaria. The biomarker score for each patient was calculated as detailed in the text. (A) Biomarker scores were plotted against observed probability of death. The two variables were significantly related (Spearman's rho = 0.96, p = 0.003). (B) Biomarker score distributions were plotted for severe malaria survivors and fatalities. (C) A receiver operating characteristic (ROC) curve was generated for the biomarker score. The dashed reference line represents the ROC curve for a test with no discriminatory ability. Area under the ROC curve is displayed on each graph with 95% confidence intervals in parentheses. *** p<0.001.

Biomarker score was highly positively correlated with risk of death (Fig. 4A; Spearman's rho = 0.96, p = 0.003). Scores were elevated among fatalities compared to survivors (Fig. 4B; median (interquartile range): 5 (4–6) and 1 (0–2.5), respectively). In a univariate logistic regression model, the biomarker score was a significant predictor of death with an odds ratio of 7.9 (95% CI 4.6–54.4) (Table 3, Model 1). After adjustment for parasitemia and age, which have been associated with malaria mortality, the score remained significant with an adjusted odds ratio of 7.8 (4.7–134) (Table 3, Model 2).

**Table 3:** Association of biomarker score with outcome among children with severe malaria: logistic regression.^a^

								Hosmer-Lemeshow test		
	Variable	b (95% CI)	SE	Wald	df	p value	OR (95% CI)	Chi square	df	p value
Model 1^b^	Biomarker score	2.1 (1.5–4.0)	2.3	18.6	1	0.001	7.9 (4.6–54.4)	3.3	5	0.66
Model 2^c^	Biomarker score^d^	2.1 (1.6–4.9)	21.5	18.2	1	0.001	7.8 (4.7–134)	1.1	8	1.0
	Log parasitemia^e^	0.050 ((−1.1)–1.3)	2.8	0.010	1	0.91	1.1 (0.35–3.6)			
	Age	0.053 ((−0.61)–1.2)	8.5	0.052	1	0.89	1.1 (0.55–3.3)			

a The reference category was “survival.”

b Pseudo-R^2^ (Cox & Snell) 0.473 and calibration slope 0.98.

c Pseudo-R^2^ (Cox & Snell) 0.474 and calibration slope 1.0.

d Biomarker score and log parasitemia had a significant but low correlation (Spearman's rho 0.292, p<0.01).

e Parasitemia was log-transformed in order to achieve linearity with the log-odds of the dependent variable. SE, standard error; OR, odds ratio.

ROC curve analysis and cut-point determination were performed as above for the biomarker score. The AUC was 0.96 (0.90–0.99) (Fig. 4C), and we found that a score ≥4 was 95.7% sensitive and 88.8% specific for predicting death in our sample (Table 4, row 1). While the positive predictive value was low (33.9%) given a fatality rate of 5.7%, the negative predictive value (NPV) was 99.7%, indicating that a child with a score ≤3 will likely respond well to standard treatment protocols.

**Table 4:** Clinical performance of biomarker combinations for predicting mortality among children with severe malaria.^a^

Combination	Cut-point^b^	Sensitivity (%)	Specificity (%)	PLR^c^	NLR	PPV (%)^d^	NPV (%)
Biomarker score (6 markers)	≥4	95.7 (78.1–99.9)	88.8 (79.7–94.7)	8.5 (7.6–9.6)	0.05 (0.007–0.4)	33.9 (12.8–61.3)	99.7 (95.2–100)
Ang-2, PCT, sICAM-1	≥2	91.3 (72.0–98.9)	88.8 (79.7–94.7)	8.1 (7.0–9.4)	0.1 (0.02–0.4)	32.9 (12.1–60.3)	99.4 (94.7–100)
Ang-2, IP-10, PCT	≥2	91.3 (72.0–98.9)	86.3 (76.7–92.9)	6.6 (5.7–7.7)	0.1 (0.02–0.4)	28.6 (10.2–54.4)	99.4 (94.6–100)
PCT, IP-10, sTREM-1	≥2	91.3 (72.0–98.9)	81.3 (71.0–89.1)	4.9 (4.1–5.7)	0.1 (0.03–0.4)	22.7 (8.1–44.8)	99.4 (94.2–100)

a All parameters are presented with 95% CIs in parentheses.

b Cut-points were determined using the Youden Index (J = max[sensitivity+specificity−1]).

c PLR, positive likelihood ratio; NLR, negative likelihood ratio; PPV, positive predictive value; NPV, negative predictive value.

d PPVs and NPVs were based on estimates that 5.7% of CM and SMA patients at Mulago hospital die of the malaria infection [28].

A score involving fewer biomarkers might be expected to improve practicality and facilitate potential translation to a clinical application. Using the same scoring scheme, 2-marker combinations performed poorly (data not shown). However, specific 3-marker combinations yielded sensitivity>90% and specificity>80% (Table 4).

### Predicting mortality using classification tree analysis

**Figure 5:**
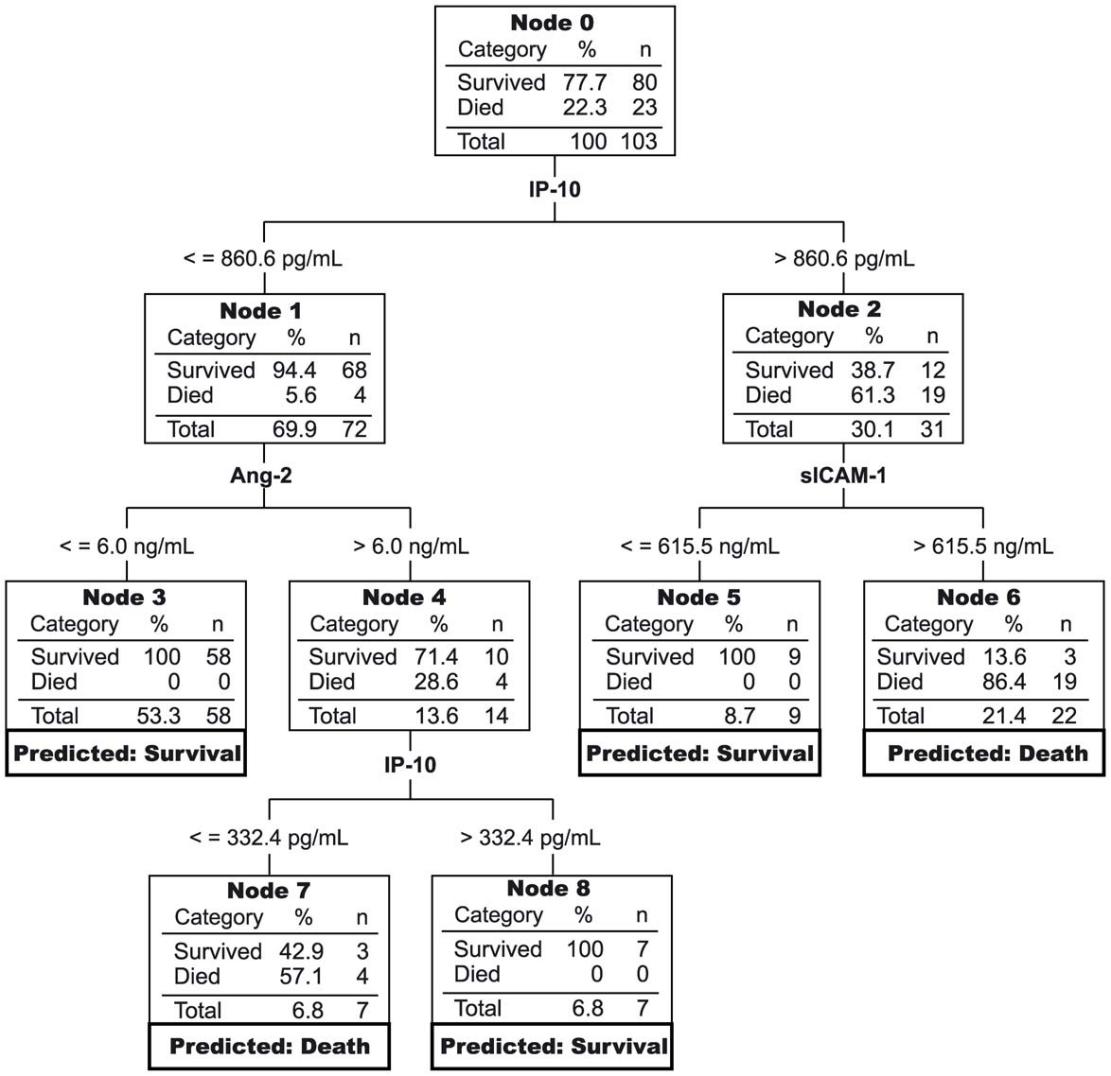
Classification tree analysis to predict outcome of severe malaria infection with host biomarkers. All six biomarkers that discriminated survivors from fatalities were entered into the classification tree analysis. Prior probabilities of survival and death were specified (94.3% and 5.7%, respectively). The cost of misclassifying a true death was designated as 10 times the cost of misclassifying a true survivor. The cut-points selected by the analysis are indicated between parent and child nodes. Below each terminal node (i.e. no further branching), the predicted categorization of all patients in that node is indicated. This model yielded 100% sensitivity and 92.5% specificity for predicting mortality (cross-validated misclassification rate 15.4% with standard error 4.9%).

To explore another combinatorial strategy, we used classification tree analysis, which selects and organizes independent variables into a decision tree that optimally predicts the dependent measure. Initially, a model based on IP-10 and sTREM-1 was generated with 43.5% sensitivity and 100% specificity for predicting mortality (data not shown). Since high sensitivity would be a crucial feature of a prognostic test for severe malaria, we repeated the analysis assigning the cost of misclassifying a death as a survivor as 10 times greater than the cost of misclassifying a survivor as a death. A model based on IP-10, Ang-2, and sICAM-1 was generated (Fig. 5), with 100% sensitivity and 92.5% specificity for predicting outcome (cross-validated misclassification rate 15.4%, standard error 4.9%). In summary, combining dichotomized biomarkers using a scoring system or a classification tree predicted severe malaria mortality in our patient population with high accuracy.

## Discussion

Combinations of prognostic biomarkers, particularly if drawn from distinct pathobiological pathways, have been found to improve predictive accuracy [35]. In this study, we demonstrated that simple schemes combining as few as 3 host biomarkers of inflammation and endothelial activation predicted mortality with high accuracy among a group of Ugandan children with severe malaria. These findings provide support for the development of prognostic tests for severe malaria based on host biomarker combinations. Moreover, we further characterized WPB exocytosis in malaria infection and identified sTREM-1 and sFlt-1 as novel biomarkers of severe and fatal malaria in children, leading to new hypotheses regarding severe malaria pathogenesis.

We found that plasma sTREM-1 levels reflected disease severity in children with severe malaria. The TREM-1 receptor is expressed on the cell membrane of monocytes and neutrophils and mediates pro-inflammatory responses [37]. sTREM-1 is generated by cleavage of membrane TREM-1 upon myeloid cell activation [38]. Both membrane and soluble TREM-1 are increased in inflammatory pathologies in humans [15], and inhibition of TREM-1 improves outcome in murine models of sepsis and inflammatory bowel disease [39], [40]. Interestingly, a recent report demonstrated increased levels of monocyte TREM-1 in uncomplicated malaria cases compared to uninfected individuals [41]. Together, these findings raise the possibility that TREM-1 may contribute to the excessive inflammation characteristic of severe malaria.

sFlt-1 is generated by alternative splicing of VEGF receptor-1 mRNA and antagonizes the pro-inflammatory and pro-angiogenic effects of VEGF. Our observation of increased sFlt-1 in severe malaria parallels findings in sepsis patients [42]. Data from murine models of sepsis suggest that sFlt-1 may have a protective role in this disease, as sFlt-1 administration reduced VEGF-mediated vascular permeability and mortality [43]. VEGF expression in the brain was increased in European travellers who died of CM compared to controls with non-neurological causes of death [44], and plasma VEGF levels positively correlated with neurological complications in African children with CM [45]. Thus, similarly to sepsis, elevated sFlt-1 in severe malaria may represent a host response to counter the pathological effects of excess VEGF. However, the role of VEGF in malaria infection is controversial: VEGF can also have neuroprotective effects, and some reports have demonstrated decreasing plasma VEGF with increasing malaria severity [24], [46]. Further studies are required to delineate the roles of sFlt-1/VEGF and sTREM-1 in severe malaria.

There were some discrepancies between our data and previous studies of biomarkers in pediatric severe malaria. s-endoglin was found to be increased in Gabonese children with severe malaria compared to UM [18], but we did not replicate these results. We observed similar levels of IP-10 in CM and SMA fatalities, in contrast to a report that serum IP-10 was specifically elevated in Ghanaian children who died from CM [14]. However, these studies may not be comparable since blood was obtained post-mortem in the Ghanaian study rather than at admission. It is also possible that the above discrepancies are due to regional differences in parasite strains, host genetics, and/or common co-infections.

As previously described [22]–[24], we observed increased plasma levels of WPB components Ang-2, vWF, and vWF propeptide in CM vs. UM. We also demonstrated for the first time that these factors are specifically elevated among children with SMA, suggesting that extensive WPB exocytosis occurs not only in CM but also in SMA. Few studies have directly addressed endothelial activation in SMA [47]. WPB exocytosis can be induced by factors generated during malaria infection (e.g., cytokines, histamine, reactive oxygen species) that have been shown to be more elevated in SMA compared to UM [9], [48]. It is biologically plausible that increased circulating levels of WPB contents could directly contribute to the pathogenesis of SMA. Ang-2 sensitization of endothelial cells to TNF [49] may amplify secretion of endothelial cytokines, such as IL-6, that can promote anemia [50]. Interestingly, Ang-2 can impair maintenance of long-term hematopoietic stem cells (LT-HSCs) in bone marrow by inhibiting the Tie-2/Ang-1 interaction [51]. While the role of LT-HSCs in SMA requires clarification, it is interesting to speculate that dysregulated Ang-2 levels may contribute to anemia via LT-HSC depletion. Unfortunately we are unable to comment on the Ang-1 levels or Ang-2/Ang-1 ratios in these children due to poor detectability of Ang-1 in citrated plasma.

Regardless of whether these biomarkers mediate or simply reflect pathology, combinations of biomarkers accurately predicted mortality among children with severe malaria in our sample. Notably, some biomarker combinations showed excellent sensitivity, ensuring that the majority of children at high risk of death would be identified. While an effective adjunctive therapy for severe malaria remains elusive, prognostication could allow triage of patients for closer monitoring or intensive care resources, as available. Such a test may also assist in risk stratification and patient selection for clinical trials of adjunctive therapies, which are ongoing [52], [53].

Previous studies have developed clinical scores to prognosticate outcome in pediatric severe malaria [3], [4]. These scores incorporate clinical features such as prostration, coma, and respiratory distress. The simplicity and low costs of these tests are attractive features. However, a prognostic assay would ideally predict mortality with both high sensitivity and specificity based on a single criterion to avoid the uncertainty associated with non-extreme scores. The biomarker combinatorial strategies presented here appear to possess this attribute, although further studies are required to confirm our findings. Another advantage of a biomarker-based prognostic test over clinical assessment is its objective quality that is unaffected by between-clinician variability. Furthermore, advances in point-of-care platforms [54] may enable development of affordable tests that integrate malaria diagnostics with prognostic biomarkers.

The limitations to our study include a small sample size and the use of non-consecutive samples, which may have introduced a selection bias. Co-infections with pathogens other than HIV were not assessed, and thus it is unclear how they may have affected biomarker levels. Biomarker combinations that accurately predicted mortality require validation in larger prospective studies with adjustment for potential demographic and clinical confounders and head-to-head comparison with prognostic clinical scores. Furthermore, these combinations require validation across different ethnicities and malaria endemicities, as well as in children with non-CM/non-SMA severe malaria syndromes. Nevertheless, this study identified novel biomarkers in African children, who are at the greatest risk of malaria mortality, and specifically in SMA, for which few informative biomarkers have been described. We provide proof-of-concept that combining as few as 3 biomarkers using simple schemes may be able to accurately predict outcome in severe malaria infection.

## References

[1] World Health Organization (2009). World Malaria Report.. http://whqlibdoc.who.int/publications/2009/9789241563901_eng.pdf.

[2] Murphy SC, Breman JG (2001). Gaps in the childhood malaria burden in Africa: cerebral malaria, neurological sequelae, anemia, respiratory distress, hypoglycemia, and complications of pregnancy.. Am J Trop Med Hyg.

[3] Marsh K, Forster D, Waruiru C, Mwangi I, Winstanley M (1995). Indicators of life-threatening malaria in African children.. N Engl J Med.

[4] Helbok R, Kendjo E, Issifou S, Lackner P, Newton CR (2009). The Lambarene Organ Dysfunction Score (LODS) is a simple clinical predictor of fatal malaria in African children.. J Infect Dis.

[5] van der Heyde HC, Nolan J, Combes V, Gramaglia I, Grau GE (2006). A unified hypothesis for the genesis of cerebral malaria: sequestration, inflammation and hemostasis leading to microcirculatory dysfunction.. Trends Parasitol.

[6] Clark IA, Alleva LM, Mills AC, Cowden WB (2004). Pathogenesis of malaria and clinically similar conditions.. Clin Microbiol Rev.

[7] Faille D, El-Assaad F, Alessi MC, Fusai T, Combes V (2009). Platelet-endothelial cell interactions in cerebral malaria: the end of a cordial understanding.. Thromb Haemost.

[8] Lyke KE, Burges R, Cissoko Y, Sangare L, Dao M (2004). Serum levels of the proinflammatory cytokines interleukin-1 beta (IL-1beta), IL-6, IL-8, IL-10, tumor necrosis factor alpha, and IL-12(p70) in Malian children with severe Plasmodium falciparum malaria and matched uncomplicated malaria or healthy controls.. Infect Immun.

[9] Othoro C, Lal AA, Nahlen B, Koech D, Orago AS (1999). A low interleukin-10 tumor necrosis factor-alpha ratio is associated with malaria anemia in children residing in a holoendemic malaria region in western Kenya.. J Infect Dis.

[10] Grau GE, Taylor TE, Molyneux ME, Wirima JJ, Vassalli P (1989). Tumor necrosis factor and disease severity in children with falciparum malaria.. N Engl J Med.

[11] Kurtzhals JA, Adabayeri V, Goka BQ, Akanmori BD, Oliver-Commey JO (1998). Low plasma concentrations of interleukin 10 in severe malarial anaemia compared with cerebral and uncomplicated malaria.. Lancet.

[12] Gyan B, Kurtzhals JA, Akanmori BD, Ofori M, Goka BQ (2002). Elevated levels of nitric oxide and low levels of haptoglobin are associated with severe malarial anaemia in African children.. Acta Trop.

[13] Chiwakata CB, Manegold C, Bonicke L, Waase I, Julch C (2001). Procalcitonin as a parameter of disease severity and risk of mortality in patients with Plasmodium falciparum malaria.. J Infect Dis.

[14] Armah HB, Wilson NO, Sarfo BY, Powell MD, Bond VC (2007). Cerebrospinal fluid and serum biomarkers of cerebral malaria mortality in Ghanaian children.. Malar J.

[15] Ford JW, McVicar DW (2009). TREM and TREM-like receptors in inflammation and disease.. Curr Opin Immunol.

[16] Beare NA, Harding SP, Taylor TE, Lewallen S, Molyneux ME (2009). Perfusion abnormalities in children with cerebral malaria and malarial retinopathy.. J Infect Dis.

[17] Medana IM, Turner GD (2006). Human cerebral malaria and the blood-brain barrier.. Int J Parasitol.

[18] Dietmann A, Helbok R, Lackner P, Fischer M, Reindl M (2009). Endoglin in African children with Plasmodium falciparum malaria: a novel player in severe malaria pathogenesis?. J Infect Dis.

[19] Turner GD, Ly VC, Nguyen TH, Tran TH, Nguyen HP (1998). Systemic endothelial activation occurs in both mild and severe malaria. Correlating dermal microvascular endothelial cell phenotype and soluble cell adhesion molecules with disease severity.. Am J Pathol.

[20] Muehlenbachs A, Fried M, Lachowitzer J, Mutabingwa TK, Duffy PE (2008). Natural selection of FLT1 alleles and their association with malaria resistance in utero.. Proc Natl Acad Sci U S A.

[21] Lowenstein CJ, Morrell CN, Yamakuchi M (2005). Regulation of Weibel-Palade body exocytosis.. Trends Cardiovasc Med.

[22] Hollestelle MJ, Donkor C, Mantey EA, Chakravorty SJ, Craig A (2006). von Willebrand factor propeptide in malaria: evidence of acute endothelial cell activation.. Br J Haematol.

[23] Lovegrove FE, Tangpukdee N, Opoka RO, Lafferty EI, Rajwans N (2009). Serum angiopoietin-1 and -2 levels discriminate cerebral malaria from uncomplicated malaria and predict clinical outcome in African children.. PLoS One.

[24] Yeo TW, Lampah DA, Gitawati R, Tjitra E, Kenangalem E (2008). Angiopoietin-2 is associated with decreased endothelial nitric oxide and poor clinical outcome in severe falciparum malaria.. Proc Natl Acad Sci U S A.

[25] Parikh SM, Mammoto T, Schultz A, Yuan HT, Christiani D (2006). Excess circulating angiopoietin-2 may contribute to pulmonary vascular leak in sepsis in humans.. PLoS Med.

[26] Bridges DJ, Bunn J, van Mourik JA, Grau G, Preston RJ (2010). Rapid activation of endothelial cells enables Plasmodium falciparum adhesion to platelet-decorated von Willebrand factor strings.. Blood.

[27] Conroy AL, Lafferty EI, Lovegrove FE, Krudsood S, Tangpukdee N (2009). Whole blood angiopoietin-1 and -2 levels discriminate cerebral and severe (non-cerebral) malaria from uncomplicated malaria.. Malar J.

[28] Opoka RO, Xia Z, Bangirana P, John CC (2008). Inpatient mortality in children with clinically diagnosed malaria as compared with microscopically confirmed malaria.. Pediatr Infect Dis J.

[29] Ministry of Health of Uganda (2006). National Policy on Malaria Treatment.. http://www.health.go.ug/mcp/NationalPolicyonMalariaTreatment(07_03_06).pdf.

[30] DeLong ER, DeLong DM, Clarke-Pearson DL (1988). Comparing the areas under two or more correlated receiver operating characteristic curves: a nonparametric approach.. Biometrics.

[31] Steyerberg EW, Eijkemans MJ, Harrell FE, Habbema JD (2001). Prognostic modeling with logistic regression analysis: in search of a sensible strategy in small data sets.. Med Decis Making.

[32] Hosmer DW, Lemeshow S (2000). Applied Logistic Regression. 2nd ed.

[33] World Health Organization (2000). Severe falciparum malaria.. Trans R Soc Trop Med Hyg.

[34] Harrell FE, Lee KL, Mark DB (1996). Multivariable prognostic models: issues in developing models, evaluating assumptions and adequacy, and measuring and reducing errors.. Stat Med.

[35] Morrow DA, Braunwald E (2003). Future of biomarkers in acute coronary syndromes: moving toward a multimarker strategy.. Circulation.

[36] Vinueza CA, Chauhan SP, Barker L, Hendrix NW, Scardo JA (2000). Predicting the success of a trial of labor with a simple scoring system.. J Reprod Med.

[37] Bouchon A, Dietrich J, Colonna M (2000). Cutting edge: inflammatory responses can be triggered by TREM-1, a novel receptor expressed on neutrophils and monocytes.. J Immunol.

[38] Gomez-Pina V, Soares-Schanoski A, Rodriguez-Rojas A, Del Fresno C, Garcia F (2007). Metalloproteinases shed TREM-1 ectodomain from lipopolysaccharide-stimulated human monocytes.. J Immunol.

[39] Bouchon A, Facchetti F, Weigand MA, Colonna M (2001). TREM-1 amplifies inflammation and is a crucial mediator of septic shock.. Nature.

[40] Schenk M, Bouchon A, Seibold F, Mueller C (2007). TREM-1–expressing intestinal macrophages crucially amplify chronic inflammation in experimental colitis and inflammatory bowel diseases.. J Clin Invest.

[41] Chimma P, Roussilhon C, Sratongno P, Ruangveerayuth R, Pattanapanyasat K (2009). A distinct peripheral blood monocyte phenotype is associated with parasite inhibitory activity in acute uncomplicated Plasmodium falciparum malaria.. PLoS Pathog.

[42] Shapiro NI, Yano K, Okada H, Fischer C, Howell M (2008). A prospective, observational study of soluble FLT-1 and vascular endothelial growth factor in sepsis.. Shock.

[43] Yano K, Liaw PC, Mullington JM, Shih SC, Okada H (2006). Vascular endothelial growth factor is an important determinant of sepsis morbidity and mortality.. J Exp Med.

[44] Deininger MH, Winkler S, Kremsner PG, Meyermann R, Schluesener HJ (2003). Angiogenic proteins in brains of patients who died with cerebral malaria.. J Neuroimmunol.

[45] Casals-Pascual C, Idro R, Gicheru N, Gwer S, Kitsao B (2008). High levels of erythropoietin are associated with protection against neurological sequelae in African children with cerebral malaria.. Proc Natl Acad Sci U S A.

[46] Jain V, Armah HB, Tongren JE, Ned RM, Wilson NO (2008). Plasma IP-10, apoptotic and angiogenic factors associated with fatal cerebral malaria in India.. Malar J.

[47] Tchinda VH, Tadem AD, Tako EA, Tene G, Fogako J (2007). Severe malaria in Cameroonian children: correlation between plasma levels of three soluble inducible adhesion molecules and TNF-alpha.. Acta Trop.

[48] Greve B, Kremsner PG, Lell B, Luckner D, Schmid D (2000). Malarial anaemia in African children associated with high oxygen-radical production.. Lancet.

[49] Fiedler U, Reiss Y, Scharpfenecker M, Grunow V, Koidl S (2006). Angiopoietin-2 sensitizes endothelial cells to TNF-alpha and has a crucial role in the induction of inflammation.. Nat Med.

[50] Raj DS (2009). Role of interleukin-6 in the anemia of chronic disease.. Semin Arthritis Rheum.

[51] Gomei Y, Nakamura Y, Yoshihara H, Hosokawa K, Iwasaki H (2010). Functional differences between two Tie2 ligands, angiopoietin-1 and -2, in regulation of adult bone marrow hematopoietic stem cells.. Exp Hematol.

[52] Yeo TW, Lampah DA, Gitawati R, Tjitra E, Kenangalem E (2007). Impaired nitric oxide bioavailability and L-arginine reversible endothelial dysfunction in adults with falciparum malaria.. J Exp Med.

[53] Boggild AK, Krudsood S, Patel SN, Serghides L, Tangpukdee N (2009). Use of peroxisome proliferator-activated receptor gamma agonists as adjunctive treatment for Plasmodium falciparum malaria: a randomized, double-blind, placebo-controlled trial.. Clin Infect Dis.

[54] Lee WG, Kim YG, Chung BG, Demirci U, Khademhosseini A (2009). Nano/Microfluidics for diagnosis of infectious diseases in developing countries.. Adv Drug Deliv Rev.

